# Assessing the Intestinal Microbiota in the SHINE Trial

**DOI:** 10.1093/cid/civ850

**Published:** 2015-11-11

**Authors:** Ethan K. Gough, Andrew J. Prendergast, Kuda E. Mutasa, Rebecca J. Stoltzfus, Amee R. Manges

**Affiliations:** 1 Department of Epidemiology, Biostatistics and Occupational Health, McGill University, Montreal, Quebec, Canada; 2 Zvitambo Institute for Maternal and Child Health Research, Harare, Zimbabwe; 3 Blizard Institute, Queen Mary University of London, United Kingdom; 4 Division of Nutritional Sciences, Cornell University, Ithaca, New York; 5 School of Population and Public Health, University of British Columbia, Vancouver, Canada

**Keywords:** microbiota, stunting, infant, environmental enteric dysfunction

## Abstract

Advances in DNA sequencing technology now allow us to explore the dynamics and functions of the microbes that inhabit the human body, the microbiota. Recent studies involving experimental animal models suggest a role of the gut microbiota in growth. However, the specific changes in the human gut microbiota that contribute to growth remain unclear, and studies investigating the gut microbiota as a determinant of environmental enteric dysfunction (EED) and child stunting are lacking. In this article, we review the evidence for a link between the developing infant gut microbiota, infant feeding, EED, and stunting, and discuss the potential causal pathways relating these variables. We outline the analytic approaches we will use to investigate these relationships, by capitalizing on the longitudinal design and randomized interventions of the Sanitation Hygiene Infant Nutrition Efficacy trial in Zimbabwe.

A key hypothesis of the Sanitation Hygiene Infant Nutrition Efficacy (SHINE) trial is that a subclinical intestinal pathology, termed environmental enteric dysfunction (EED), underlies child stunting [1]. A relatively unexplored factor in the etiology of EED and linear growth failure is the gut microbiota, which critically impacts nutrient utilization, intestinal structure and function, immune development, and systemic inflammation in early life. Infant gut microbiota development is influenced by childbirth (via vertical transmission of the maternal microbiota), diet, and fecal–oral exposure to enteric pathogens in conditions of poor sanitation and hygiene. The interdependence between infants and their earliest symbionts is very likely to impact the growth trajectory of the child, with profound and persistent health effects throughout life.

In a substudy of the SHINE trial, we will examine how the gut microbiota contributes to linear growth between 0 and 18 months of age among human immunodeficiency virus (HIV)–unexposed infants (ie, infants born to women who tested HIV negative at baseline and at 32 gestational weeks). We hypothesize that the developing infant gut microbiota may impact child growth via 2 potential pathways: (1) a nutrition pathway, through which the gut microbiota can make nutrients and energy available to the infant (or decrease their availability); and (2) an EED pathway, through which the gut microbiota drives subclinical changes in intestinal morphology, leading to increased intestinal permeability, allowing passage of microbes and/or their byproducts across a compromised intestinal barrier (microbial translocation), which results in chronic systemic inflammation, suppression of insulin-like growth factor 1 (IGF-1), and redirection of nutrients and energy away from growth (Figure 1).

**Figure 1.**
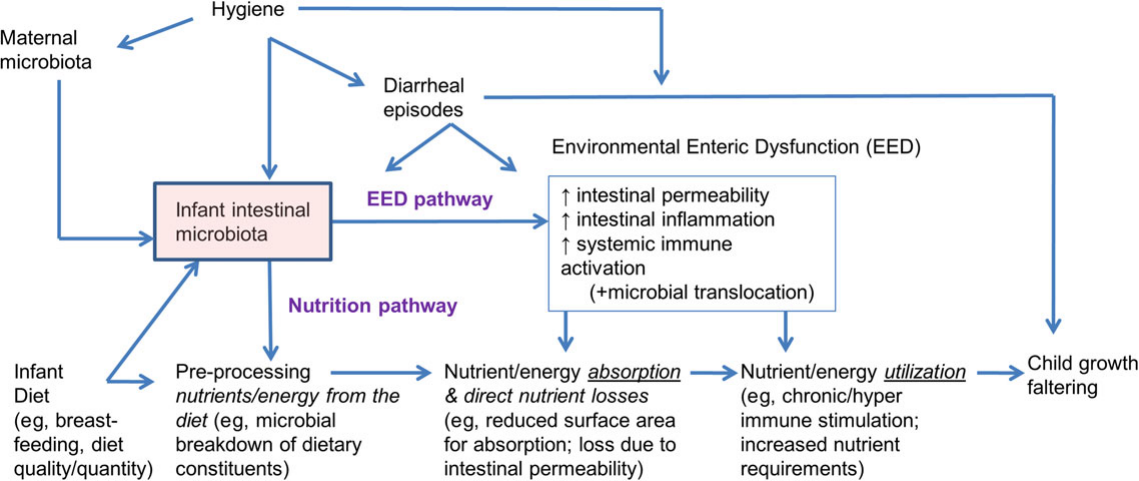
Schematic of the 2 hypothesized causal pathways linking the gut microbiota to infant growth faltering. Abbreviation: EED, environmental enteric dysfunction.

## EVIDENCE THAT THE GUT MICROBIOTA IS ASSOCIATED WITH INFANT GROWTH

Historical evidence has shown that modulating the gut microbiota with antimicrobials leads to improved weight gain and feed efficiency in food animals [2]. A meta-analysis of 10 randomized controlled trials from low- and middle-income countries, representing a variety of antibiotic treatments, showed that, on average, antibiotic use increased height and weight in prepubertal children [3], consistent with observational findings from developed countries [4]. Randomized controlled trials of prebiotic or probiotic interventions have also found significant weight gain in infants fed formula milk containing *Bifidobacterium lactis* (a probiotic microorganism believed to provide health benefits when consumed) or containing fructo- or galacto-oligosaccharides (prebiotic carbohydrates that promote the growth of normally abundant, beneficial microbes in the human intestine), compared with infants given control formula [5, 6]. The microbiota has been shown to be important in bone density [7, 8], and the use of antimicrobials leads to alterations in microbiota composition [9–11], increased serum IGF-1, and increased growth (length and weight) in animals [12–14]. IGF-1 mediates the effects of growth hormone and thereby directly controls linear growth. Observational studies [15–18] demonstrate a relationship between the configuration of the gut microbiota and severe acute malnutrition in children. Transplantation of donor feces from children with severe acute malnutrition, but not their healthy twins, led to weight loss in gnotobiotic mice fed a typical Malawian diet [19], while increases in total body mass and fat mass were induced in mice transplanted with donor feces from obese adults, but not their lean twins [20]. Taken together, this evidence suggests a causal effect of the microbiota on growth, particularly for weight, although the specific changes that impact growth are unknown.

The infant microbiota, which is vertically transmitted from mother to infant at birth and via breastfeeding, may influence the initial course of early postnatal growth. Infant gut microbiota composition is related to the maternal gut microbiota shortly after birth and then diverges [21, 22], and mode of delivery can influence the early microbiota. Vaginally delivered newborns appear to harbor a gut microbiota similar to the maternal vaginal microbiota, while the microbiota of newborns delivered by cesarean method more closely resemble the microbiota of the maternal skin and surrounding environment. *Lactobacillus*, *Prevotella*, or *Sneathia* species predominate in vaginally delivered infants [23]. The gut microbiota composition tends to be individual-specific, suggesting that the initial colonizing bacteria play a pivotal role in how the microbiota develops [24, 25]. Age-specific succession of bifidobacterial species has been observed in healthy infants from 0 to 24 months of age and in pregnant mothers [26, 27]. The development and assembly of the infant gut microbiota correlate temporally with the period of greatest infant growth faltering.

## GUT MICROBIOTA, EED, AND INFANT GROWTH FALTERING

We recently showed that stunting in Zimbabwean infants is characterized by low-grade chronic inflammation, which is apparent as early as 6 weeks of age and is associated with lower levels of IGF-1. Levels of intestinal fatty acid binding protein (I-FABP), indicating damage to small intestinal villi, were also markedly elevated compared with healthy European children [28]. A number of other studies have shown that inflammation and permeability of the small intestine are associated with poor linear growth [29–33]. Taken together, these findings suggest that an extensive enteropathy occurs during infancy and that stunting may be an inflammatory disease. Early microbiota assembly and composition via environmental exposures is thought to be a critical factor in the development and pathogenesis of EED [34]. Gut microbiota alteration is also implicated in the pathogenesis of other inflammatory enteric diseases (eg, inflammatory bowel disease, irritable bowel syndrome, and celiac disease).

There is a complex relationship between environmental exposures, including colonization and infection with enteric pathogens, and assembly of the infant gut microbiota. Enteric pathogen exposure may impair healthy microbiota assembly during infancy. Up to 80% of infants living in unsanitary conditions, and who are not exclusively breastfed, may be infected with at least 1 enteric pathogen by age 6 months [35], indicating exposure to potentially pathogenic organisms very early in life; furthermore, enteric pathogen carriage is often asymptomatic [36]. Formative work in rural Zimbabwe demonstrated that geophagia and consumption of chicken feces is common in infancy, and that these exposures are associated with a high burden of enteropathogens [37].

Recent reports have linked disturbances in the gut microbiota to changes in innate and adaptive immune responses [38–41] and to alterations in intestinal permeability [40, 42, 43]. Specific organisms and functional microbial products may influence susceptibility to EED. Examples include the immunomodulatory *Bacteroides fragilis* polysaccharide A [44, 45], the antimicrobials produced by *Lactobacillus reuteri* [46–49], the expression of tight junction–associated protein zonula occludens 1 by other lactobacilli [47, 48], and butyrate-producing species that increase colonization resistance by lowering pH and that enhance intestinal barrier function [50–54]. Specific pathogens such as *Citrobacter rodentium* have been associated with reduced villus height and increased intestinal permeability (both markers of EED) in Zambian adults [55]. Gut microbes that may impact glutamate levels (a key metabolite in gut amino acid metabolism) were associated with future linear growth deficits in infants in a recent analysis [56]. It is not yet known whether mothers who have EED harbor a distorted microbiota or whether an EED-susceptible microbiota exists and can be passed to the infant.

Diarrhea prevention has been another target of interventions to restore linear growth deficits [1]. There are only a few studies investigating the gut microbiota in young children with diarrhea; none specifically addresses the impact of diarrhea on the microbiota and subsequent stunting. Substantial reductions in age-associated microbiota diversity have been associated with diarrhea in children [18, 57]. Pop and colleagues found expected pathogens, as well as unexpected microbiota members, to be associated with moderate to severe diarrhea. They also observed shifts in anaerobic microbial taxa and genus-specific changes in *Escherichia/Shigella* and *Prevotella* between diarrheal cases and controls [57].

## GUT MICROBIOTA, NUTRITION, AND INFANT GROWTH FALTERING

The composition and function of the gut microbiota can impact the efficiency of nutrient extraction from breast milk and other foods leading to poor growth. The primary constituents of human milk include lipids, lactose, and protein. Infants can also digest host-associated glycans such as cellulose and mucin. The microbiota transitions from an early community just after birth [58] to communities characterized by *Lactobacillus* species, *Clostridium* species, *Veillonella* species, and certain *Bifidobacterium* species, which can metabolize breast milk oligosaccharides [59]. Breast milk oligosaccharides are important for healthy growth [60–62] and may selectively nourish compatible microorganisms crucial to infant and child health [63], such as bifidobacteria. Different breastfeeding patterns and early introduction of non-breast-milk foods may modulate the gut microbiota and influence postnatal growth through nutrition and EED pathways.

The introduction of complementary foods has been shown to radically alter the infant intestinal community, leading to a mature, adult-like microbiota [59, 64]. The importance of diet on gut microbiota composition was demonstrated in a comparative study of children in Burkina Faso and Italy [65], which showed that African infants exposed to a high-fiber diet were enriched for *Bacteroides*, whereas European infants exposed to a high-calorie diet were enriched for *Firmicutes*. Transplantation of stool from Malawian children with kwashiorkor into gnotobiotic mice resulted in significant weight loss in recipient mice compared with mice receiving a healthy child's microbiota; however, weight loss occurred only in the mice receiving the nutrient-poor Malawian diet vs standard chow, indicating that the microbiota growth effect is modified by diet. Blooms of lactobacilli and bifidobacteria were observed when gnotobiotic mice, which were transplanted with stool from a child with kwashiorkor, were switched from a Malawian diet to a therapeutic food (a peanut butter formulation). Administration of the therapeutic food resulted in immediate weight gain and radical changes in the microbiota, and was shown to improve the metabolic function of the gut microbiota [19].

## MICROBIOTA INVESTIGATIONS IN THE SHINE TRIAL

The SHINE trial will provide a platform to examine the evolution of the gut microbiota in response to diet, infection, and environmental exposures, and its contribution to differences in growth in infancy. The factorial design of SHINE enables us to evaluate the impact of water, sanitation, and hygiene (WASH) and infant and young child feeding (IYCF) interventions on the microbiota and linear growth. In the WASH arms, the package of interventions is expected to reduce enteropathogen burden, promote healthy microbiota assembly, and improve linear growth. In the IYCF arms, Nutributter is expected to elicit a dramatic change in the microbiota, may lead to a recovery in microbiota function, and may rescue the growth of some infants [66].

We propose to study the infant gut microbiota by performing shotgun DNA (metagenomic) and RNA (metatranscriptomic) whole-genome sequencing from fecal samples collected at 1, 3, 6, 12, and 18 months of age in a random sample of HIV-unexposed SHINE infants included in the EED substudy [67], with equal numbers of infants from the WASH and IYCF arms. The microbiota from a subgroup of mothers will be evaluated at 30–34 gestational weeks and at the 1-month postnatal visit. Changes in the microbiota will be more complex for HIV-exposed infants because they will receive cotrimoxazole prophylaxis from 6 weeks of age [68]; these infants will therefore be investigated separately.

**Figure 2.**
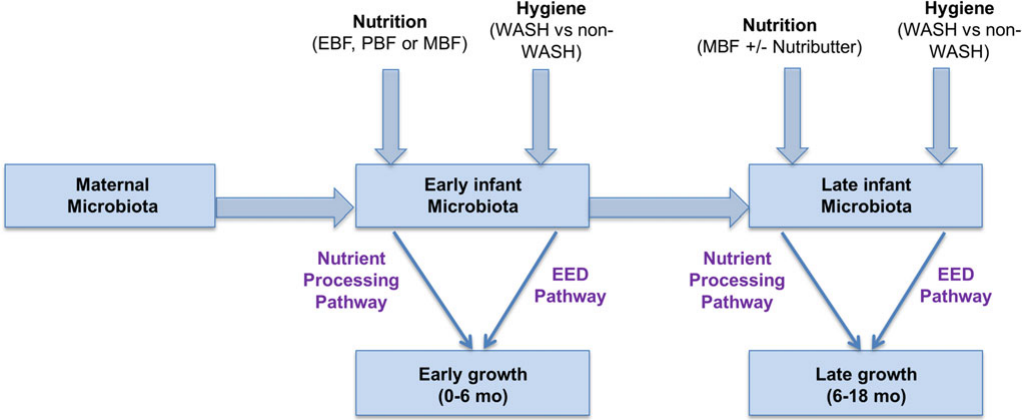
Schematic illustrating the analysis plan for the investigation of the microbiota in early and late infant growth as part of the Sanitation Hygiene Infant Nutrition Efficacy (SHINE) trial. Abbreviations: EBF, exclusive breastfeeding; EED, environmental enteric dysfunction; MBF, mixed breastfeeding [69]; PBF, partial breastfeeding; WASH, water, sanitation, and hygiene intervention.

Our substudy organizes the research questions and aims according to developmentally relevant stages of infant development (Figure 2). First, we will examine early exposures leading to the establishment of the infant gut microbiota and early growth faltering between 0 and 6 months. We will look specifically at the role of vertical transmission of the maternal microbiota and establishment of the early infant gut microbiota. We will measure the impact of nutrition (eg, exclusive, predominant, or mixed breastfeeding) on the early gut microbiota (nutrition pathway), and we will test the impact of the randomized WASH intervention on the early gut microbiota and early infant growth between 0 and 6 months (EED pathway). Second, we will examine the late infant gut microbiota and late infancy growth faltering between 6 and 18 months. We will examine the role of the early infancy microbiota on the late infancy microbiota. We will test the impact of the randomized IYCF interventions (nutrition pathway) and WASH interventions (EED pathway) on the late gut microbiota and late infant growth. The late infancy period is also characterized by increasing infant exploratory behavior, fecal–oral exposures, and diarrheal episodes, which will result in substantial shifts in the microbiota.

## MEASURING THE GUT MICROBIOTA

We will infer microbiota composition and function from microbial whole-genome sequencing. To characterize the genetic content of the gut microbiota, DNA (or RNA) sequences will be classified as microbial taxa (composition) or orthologous gene families (function). Orthologous gene families represent genes from different species that are known to or predicted to have the same function. High-quality sequencing reads will be annotated with microbe composition and function using MetaPhlAn [70] and HUMAnN [71], respectively. Sequencing technologies and analysis tools are undergoing rapid evolution; therefore, tools currently under development may also be used. Following annotation, one dataset will contain the relative abundance for each bacterial taxon (at the lowest well-defined phylogenetic level) for each subject and time-point, and a second dataset will contain the relative abundance of each orthologous gene family (at the lowest well-defined functional level) for each subject and time-point. Each dataset will also contain information on important covariates (eg, exclusive breastfeeding, diarrhea) from the SHINE trial.

## STATISTICAL ANALYSES

Statistical analyses for this substudy are complex for the following reasons: (1) desire to have results with causal interpretation to inform new microbiota interventions and to delineate separate causal pathways (Figure 1); (2) integration of multiple types of data (microbiota “omics,” epidemiologic and clinical data); (3) analysis of high-dimensional data (ie, more variables than study subjects); (4) control of confounding (eg, diarrhea, changes to diet); and (5) minimizing false discovery.

Multivariable regression can accommodate microbiota sequencing, epidemiologic, and clinical data (eg, EED markers). However, for a single cohort, DNA sequencing and annotation can identify and quantify the presence and abundance of >1000 bacterial species and thousands of orthologous gene families [72]. It will be necessary to reduce the dimensionality of these data by preselecting important microbiota members or functions. The analyses will therefore occur in stages. The first stage will involve a reduction in data dimensionality. This will be achieved using standard measures such as α (species richness) and β (between time-points or samples) diversity measures; variable selection approaches such as penalized regression (eg, least absolute shrinkage and selection operator [LASSO]) [73], random forests [74] or other machine learning methods; and selecting taxa or functions that have high microbiota network centrality [56]. A defined false-discovery rate [75] will be used at this stage to minimize false-positive results due to multiple comparisons. Specific microbial taxa or functions identified in this data reduction stage will be selected for inclusion in multivariable regression models (stage 2). Data reduction and variable selection will reduce data complexity and allow us to identify and analyze the most influential microbiota members and functions in the second analysis stage.

The second analysis stage will involve implementing multivariable regression models informed by our causal graphs (Figures 1 and 2). Infant growth will be defined by *z* scores calculated using the World Health Organization multicountry growth standard, which was designed to assess growth adequacy in breastfed infants globally [76]. We will examine each growth interval (early or late) separately, because the effects of the microbiota and its importance relative to other factors may vary during this dynamic period of infant growth and development. We will build a series of multivariable regression models in which growth increment is the dependent variable and selected microbiota features are the exposures of interest, conditioned on the attained size at the start of the interval, infant sex, and the genetic growth potential of the infant as represented by maternal height. Diarrhea, infant feeding, WASH, and Nutributter intervention/adherence will also be considered as covariates in these models. To address the dynamic nature of the cohort and possible issues of time-varying confounding induced by changes in SHINE intervention adherence, or changes in breastfeeding and diarrheal disease, we will implement marginal structural models [77, 78]. The marginal structural model is a causal adjustment method that allows the total causal effect of a time-varying exposure to be estimated consistently. Microbiota features emerging from the stage 1 analyses will also be included and analyzed as mediators in stage 2 using marginal structural models to evaluate shifts in microbiota composition and function as intermediate steps in the nutrition and EED causal pathways to infant growth (mediation analysis) [79]. We will evaluate microbiota profiles associated with EED as a secondary end-point using the same 2-stage approach (ie, data reduction/variable selection followed by multivariable regression and marginal structural models).

## CONCLUSIONS

There is growing interest in the role of the gut microbiota in infant growth due to its role in intestinal inflammation, protection from pathogens, nutrient harvesting, and absorption in the gut. Assembly of the infant gut microbiota also correlates temporally with the period of greatest infant linear growth faltering. Understanding the role of the gut microbiota in infant growth will enable the mechanisms through which SHINE interventions operate to be better defined, and may point to additional strategies for prevention of stunting. The SHINE trial therefore offers an opportunity to quantify and elucidate the causes and potential pathways of microbiota-mediated infant growth effects in the context of a randomized experiment.
